# Risk of adverse events in elotuzumab-treated patients with multiple myeloma: a systematic review and meta-analysis

**DOI:** 10.1080/07853890.2026.2641930

**Published:** 2026-03-12

**Authors:** Zhaoyue Yan, Huali Dong, Yiping Du, Dapeng Li, Linlin Liu

**Affiliations:** a Department of Hematology, Zibo First Hospital, Zibo, Shandong, China; b Department of Hematology, Qingdao eighth people’s hospital, Qingdao, Shandong, China; c Department of Genetics and Cell Biology, School of Basic Medicine, Qingdao University, Qingdao, Shandong, China

**Keywords:** Multiple myeloma, elotuzumab, adverse events, meta-analysis

## Abstract

**Background:**

Elotuzumab, an anti-SLAMF7 monoclonal antibody for multiple myeloma (MM), lacks a comprehensive safety profile from meta-analysis.

**Methods:**

We systematically searched PubMed, Web of Science, EMBASE and CENTRAL through February 13, 2025 for randomized controlled trials (RCTs) evaluating elotuzumab in MM. Pooled relative risks(RRs) of adverse events observed in elotuzumab-containing regimens versus control therapies.

**Results:**

6 RCTs (N=1,736) were included. Elotuzumabsignificantly reduced incidence of neutropenia (RR = 0.86, 95% CI: 0.76–0.98), but increased risks of cough (RR = 1.41, 95% CI: 0.96–2.09), pneumonia (RR = 1.30, 95% CI: 1.07–1.59), diarrhea (RR = 1.16, 95% CI: 1.05–1.30), pyrexia (RR = 1.47, 95% CI: 1.10–1.96) and infections (RR = 1.09, 95% CI: 1.03–1.15). No significant differences were observed for anemia, thrombocytopenia, respiratory infections, nausea, appetite loss, back pain, muscle spasms, peripheral edema, insomnia, rash, pruritus, fatigue, or hypokalemia. For grade 3–4 events, elotuzumab was associated with higher risks of lymphopenia (RR = 1.86, 95% CI: 1.31–2.64, *p* = 0.0005, I^2^ = 9%), diarrhea (RR = 1.47, 95% CI: 1.00–2.17), pneumonia (RR = 1.57, 95% CI: 1.11–2.23), cataracts (RR = 2.87, 95% CI: 1.15–7.21) and infections (RR = 1.30, 95% CI: 1.04–1.62).

**Conclusion:**

Elotuzumab in MM appears safe but with a specific adverse events pattern : lower neutropenia, but higher respiratory, gastrointestinal, metabolic, and infectious events. Differences may be influenced by longer treatment and corticosteroid use; therefore, interpretation of outcomes such as hyperglycemia and cataracts requires particular caution.

## 1. Introduction

Multiple myeloma (MM) is a hematologic malignancy and represents the second most common blood cancer worldwide and remains a major cause of cancer-related morbidity and mortality [1]. Clinically, MM typically involves triplet regimens combining an immunomodulatory drug (IMiD), such as lenalidomide or thalidomide, or a proteasome inhibitor (PI), such as bortezomib or carfilzomib, with dexamethasone. These agents act synergistically by disrupting the tumor microenvironment, promoting apoptosis, and enhancing anti-myeloma immune responses, thereby improving overall response rates and prolonging progression-free survival (PFS) [2,3]. Nonetheless, the majority of patients eventually relapse or become refractory to therapy, with diminished responsiveness to subsequent lines of treatment. Moreover, treatment-related toxicities, especially grade 3–4 adverse events (AEs) as defined by the Common Terminology Criteria for Adverse Events (CTCAE), tend to accumulate over time, compromising tolerability and limiting long-term therapeutic benefit [4,5].

The advent of monoclonal antibody-based immunotherapies has ushered in a new era in MM management. Among these, elotuzumab, a humanized IgG1 monoclonal antibody targeting signaling lymphocytic activation molecule F7 (SLAMF7), has demonstrated clinical promise [6,7]. Elotuzumab exerts its antitumor effects primarily by enhancing antibody-dependent cellular cytotoxicity through activation of natural killer cells [8,9]. When administered in combination with lenalidomide and dexamethasone or with PIs, elotuzumab has shown synergistic efficacy and has been associated with improved PFS in several phase II and III clinical trials [10–13]. However, growing clinical experience and trial-based evidence have raised important safety concerns regarding elotuzumab use. These include hematologic toxicities such as neutropenia and thrombocytopenia, as well as increased risks of infectious complications [14–17]. Infusion-related reactions (IRRs) and viral reactivation—particularly herpes zoster—are also frequently observed [18,19]. Unlike conventional cytotoxic agents, elotuzumab may elicit a distinct spectrum of AEs due to its immunostimulatory mechanism [20]. Despite the growing clinical use of elotuzumab, comprehensive and quantitative data on its safety profile remain limited.

Here, we conducted a comprehensive systematic review and meta-analysis of randomized controlled trials investigating elotuzumab-based regimens in patients with both newly diagnosed multiple myeloma (NDMM) and RRMM. We also explored the influence of treatment regimen and patient characteristics on AE risk. By synthesizing data across studies, this analysis aims to provide an evidence-based safety profile of elotuzumab, support individualized risk–benefit assessments, and inform rational integration of this agent into MM treatment algorithms.

## 2. Materials and methods

### 2.1. Systematic literature search

This systematic review and meta-analysis were conducted in accordance with the Preferred Reporting Items for Systematic Reviews and Meta-Analyses (PRISMA) guidelines. A comprehensive search of the PubMed, Web of Science, EMBASE, and Cochrane Central Register of Controlled Trials (CENTRAL) databases was performed to identify eligible clinical trials published up to February 13, 2025. The search strategy combined Medical Subject Headings (MeSH) and free-text terms related to multiple myeloma and elotuzumab. The key search terms included: (‘Multiple Myeloma’ OR ‘Plasma Cell Myeloma’ OR ‘Kahler Disease’ OR ‘Myelomatosis’) AND (‘Elotuzumab’ OR ‘Empliciti’ OR ‘HuLuc63’ OR ‘PDL063’ OR ‘BMS-901608’). The search was restricted to English-language publications involving human subjects. Two reviewers independently conducted the database search, and discrepancies were resolved through discussion or consultation with a third reviewer.

### 2.2. Literature selection

Both NDMM and RRMM trials were included to comprehensively capture the safety profile of elotuzumab across disease stages. For trials with multiple publications, only the most recent or complete dataset was included. Two reviewers independently evaluated each study’s eligibility, and disagreements were resolved by consensus. All retrieved references were imported into EndNote software for de-duplication. Subsequently, titles and abstracts were screened for eligibility. Full-text articles were retrieved for potentially relevant studies and assessed against predefined inclusion and exclusion criteria. Studies were included if they involved: (i) Phase II or III RCTs involving adult patients (≥18 years) with a confirmed diagnosis of multiple myeloma; (ii) an intervention arm containing elotuzumab in combination with standard therapy; (iii) a comparator arm lacking elotuzumab; and (iv) quantitative reporting of treatment-emergent adverse events (TEAEs) in both elotuzumab-containing and control treatment arms were extracted from eligible trials, all of which mandated concomitant corticosteroid administration according to study protocols. Studies were excluded if they were: (i) non-clinical studies, narrative reviews, case reports, meeting abstracts, editorials, or commentaries; (ii) duplicate publications (only the version with the most complete or updated data was retained); (iii) studies involving unapproved or investigational agents in either arm, unless both groups were comparably treated; (iv) trials with very small sample sizes (<10 patients per arm), which may limit interpretability or (v) trials evaluating elotuzumab in combination with proteasome inhibitors, such as bortezomib, to reduce heterogeneity and improve interpretability.

### 2.3. Data extraction

Two reviewers independently extracted data using a pre-specified, standardized data collection form. Any discrepancies were resolved through discussion or adjudication by a third reviewer. Safety data were extracted exclusively from trials evaluating elotuzumab plus IMiD-based regimens and corresponding control arms. Extracted variables included: (i) study characteristics: first author, year of publication or conference presentation, trial phase, study acronym, and trial registration number; (ii) patient characteristics: sample size, median age, gender distribution, disease stage, or prior lines of therapy; (iii) treatment characteristics: type and dose of elotuzumab, combination agents, and treatment duration; and (iv) safety outcomes: overall incidence of AEs, hematologic and non-hematologic AEs, as well as specific adverse events such as neutropenia, infections, infusion-related reactions (IRRs), and immune-related toxicities. All elotuzumab-containing regimens mandated concomitant corticosteroid administration according to trial protocols.

### 2.4. Statistical analysis

All statistical analyses were conducted using Review Manager (RevMan) version 5.4. Cochran’s Q test (significance threshold α = 0.10) and the I^2^ statistic were used to assess heterogeneity among studies. For low heterogeneity (*p* > 0.10 and I^2^ ≤ 50%), a Mantel–Haenszel fixed-effects model was applied. For moderate to high heterogeneity (*p* ≤ 0.10 or I^2^ > 50%), a DerSimonian–Laird random-effects model was employed. The primary outcome was the relative risk (RR) of treatment-emergent adverse events in patients receiving elotuzumab-based therapy versus controls, presented with 95% confidence intervals (CIs) [21]. Subgroup and sensitivity analyses were planned to explore the impact of disease setting (NDMM vs. RRMM). The present meta-analysis evaluates relative frequencies of reported treatment-emergent adverse events (TEAEs) as provided in the original trial reports, rather than exposure-adjusted incidence rates, due to the lack of uniformly reported patient-level exposure time across studies.

## 3. Results

### 3.1. Study selection

**Figure 1.**
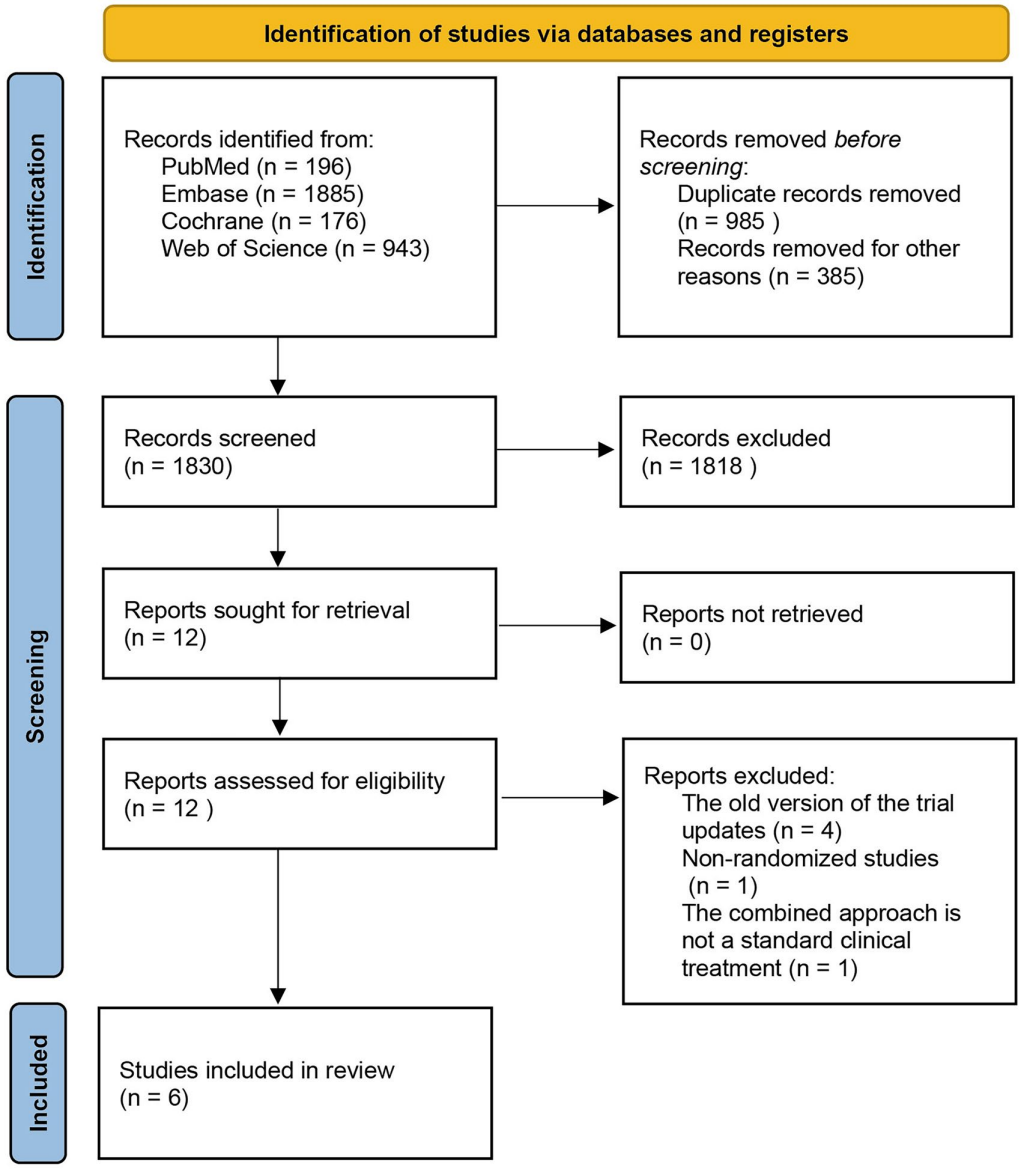
PRISMA flow diagram summarizing the systematic search, screening, and selection process for randomized controlled trials included in the meta-analysis.

A total of 3,200 records were identified through database searches, including PubMed (*n* = 196), Embase (*n* = 1,885), Cochrane Library (*n* = 176), and Web of Science (*n* = 943). After excluding 985 duplicates, 1,830 records remained for title and abstract screening. Ultimately, 12 articles underwent full-text review, of which 6 were excluded (4 earlier versions of included trials, 1 non-randomized study and 1 randomized trial evaluating elotuzumab in combination with bortezomib), yielding 6 randomized controlled trials (RCTs) for final inclusion. The study selection process is illustrated in the PRISMA flow diagram (Figure 1).

### 3.2. Quality assessment

**Figure 2.**
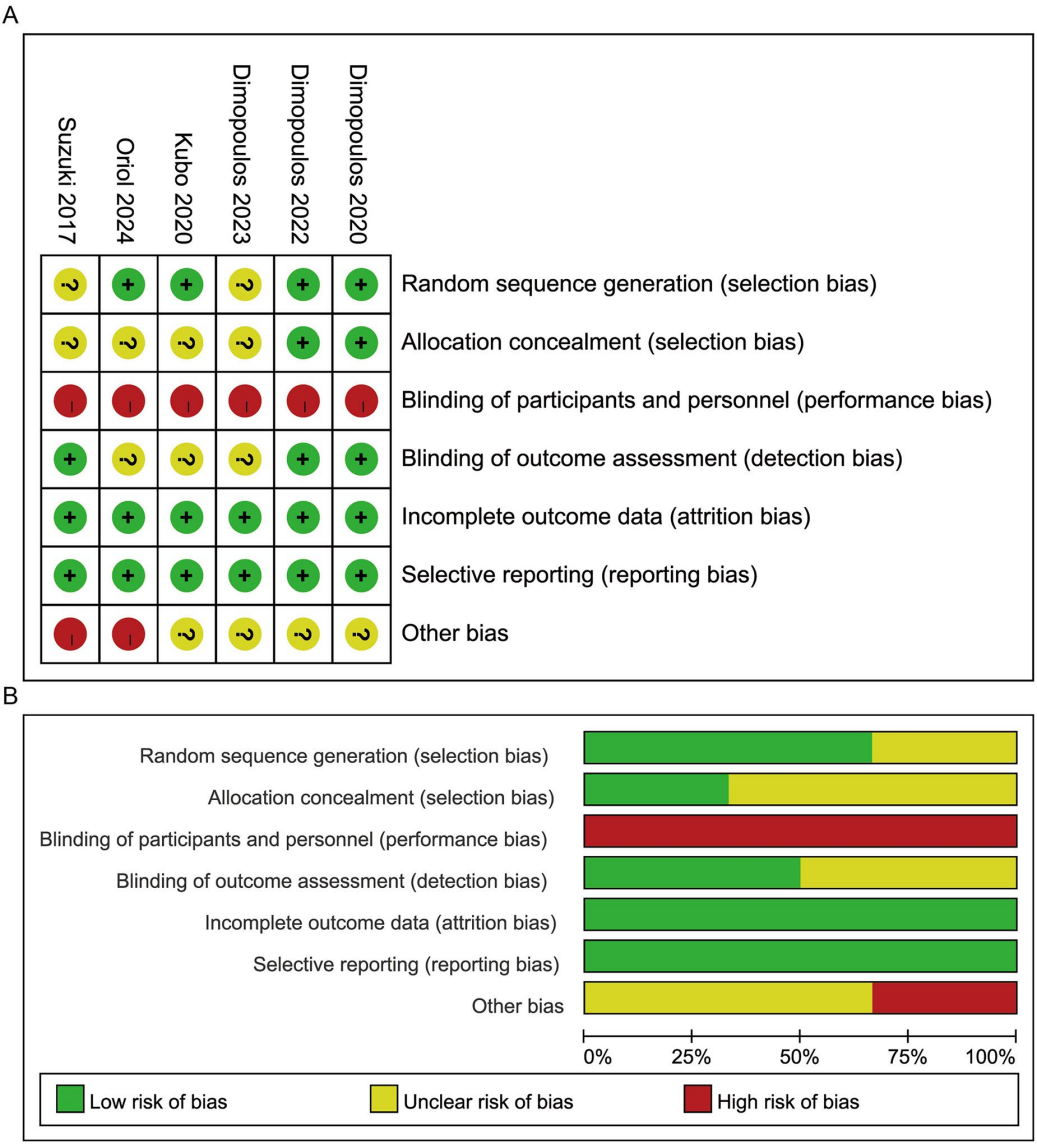
Risk of bias assessment of included randomized controlled trials using the Cochrane Risk of Bias Tool. (A) Summary of risk across all domains. (B) Domain-specific risk of bias for individual studies.

Risk of bias was assessed using the Cochrane Risk of Bias Tool within RevMan. Among the 6 included trials, 4 clearly described methods for random sequence generation [22–25]. However, allocation concealment was either unclear or unreported in 5 studies [24–27]. All studies were open-label, introducing a high risk of performance bias due to the lack of blinding of participants and investigators. Three trials reported blinding of outcome assessment [22,23,27]. All studies were judged to have a low risk of bias for incomplete outcome data and selective reporting. Detailed risk of bias assessments are presented in Figure 2.

### 3.3. Study characteristics

The 6 included RCTs comprised 4 phase III and 2 phase II trials. All analyses were based on the most recent trial versions [22–27]. A total of 1,736 patients with multiple myeloma (MM) were enrolled, including 847 in the elotuzumab-based intervention groups and 889 in the control groups. Baseline characteristics were well balanced across groups, with median ages ranging from 66 to 73 years. 2 trials specifically enrolled newly diagnosed or treatment-naïve patients [23,25], while 4 trials focused on relapsed/refractory MM, consistent with the predefined inclusion criteria [22,24,26,27]. After exclusion of the elotuzumab–bortezomib trial, the final meta-analysis included six randomized controlled trials evaluating elotuzumab in combination with immunomodulatory drugs (IMiDs). Elotuzumab was administered in combination with lenalidomide and dexamethasone in four trials [22,23, 25,27], pomalidomide and dexamethasone in two studies [24,26]. Detailed characteristics of the included trials are shown in Table 1.

**Table 1.** Summary of included trials, including study phase, patient population, intervention and comparator arms.

First author	Year of publication	Trial name	NCT identifier	Phase	Status of enrolled patients	NO. of patients^a^	Median age(years)^a^	Sex (males)^a^	Intervention treatments	Control treatments
Dimopoulos et al. [16]	2020	ELOQUENT-2	NCT01239797	3	Relapsed/ Refractory MM	318/317	67 (37–38)/66 (38–91)	NA	Elotuzumab/Lenalidomide/Dexamethasone	Lenalidomide/Dexamethasone
Dimopoulos et al. [17]	2022	ELOQUENT-1	NCT01335399	3	Newly diagnosed/ Untreated MM	371/371	73 (68–78/73(69–78)	211 /201	Elotuzumab/Lenalidomide/Dexamethasone	Lenalidomide/Dexamethasone
Dimopoulos et al. [18]	2023	ELOQUENT-3	NCT02654132	2	Relapsed/ Refractory MM	60/55	68.5 (43–81)/66 (36–82)	32/35	Elotuzumab/Pomalidomide/Dexamethasone	Pomalidomide/Dexamethasone
Kubo K et al. [19]	2020	NA	NCT02272803	2	Newly diagnosed/ Untreated MM	40/42	72 (65–83)/73 (62–89)	17 /22	Elotuzumab/Lenalidomide/Dexamethasone	Lenalidomide/Dexamethasone
Oriol A et al. [20]	2024	CheckMate 602	NCT02726581	3	Relapsed/ Refractory MM	24/72	66.5 (30–83)/68.0 (34–81)	16 /51	Nivolumab /Elotuzumab/Pomalidomide/Dexamethasone	Nivolumab /Pomalidomide/Dexamethasone
Suzuki K et al. [21]	2017	ELOQUENT-2	NCT01241292	3	Relapsed/ Refractory MM	31/29	69 (45–80)/66 (47–84)	NA	Elotuzumab/Lenalidomide/Dexamethasone	Lenalidomide/Dexamethasone

^a^ Date presented as Elotuzumab combination group vs non-Elotuzumab treatment regimen group; Abbreviations: MM: multiple myeloma, NA: not available.

### 3.4. Overall adverse events (AEs)

**Figure 3.**
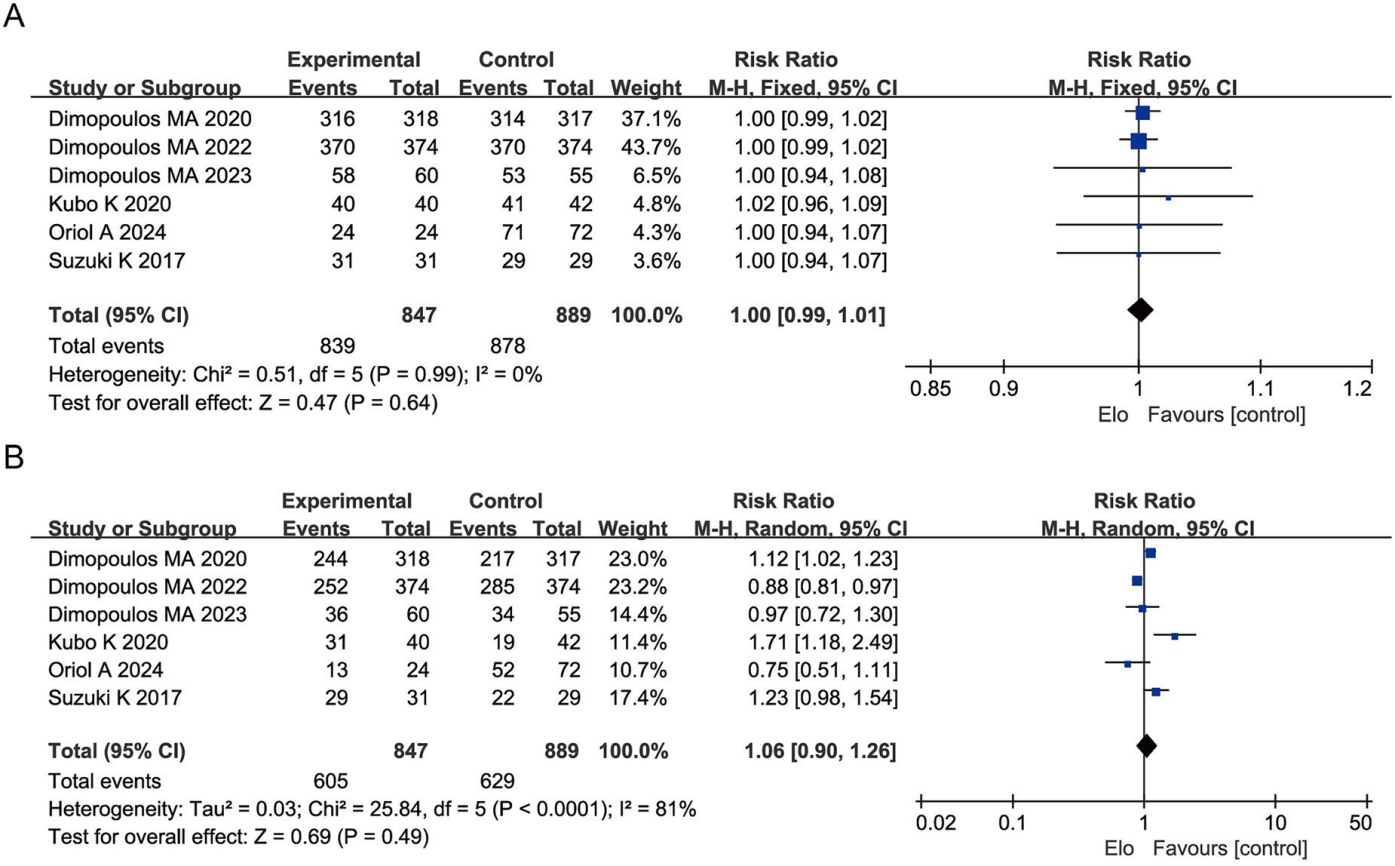
Forest plots comparing the incidence of adverse events between elotuzumab-containing and control regimens: (A) Any-grade adverse events (AEs); (B) Grade 3–4 AEs.

Pooled analysis of overall AEs from the 6 trials showed no significant difference in incidence between the elotuzumab and control groups (RR = 1.00, 95% CI: 0.99–1.01; *p* = 0.64; I^2^ = 0%, fixed-effects model) (Figure 3A). For grade 3–4 AEs, the elotuzumab group exhibited a slightly higher incidence, though the difference was not statistically significant (RR = 1.06, 95% CI: 0.90–1.26; *p* = 0.69; I^2^ = 81%, random-effects model) (Figure 3B).

### 3.5. Hematologic adverse events

**Figure 4.**
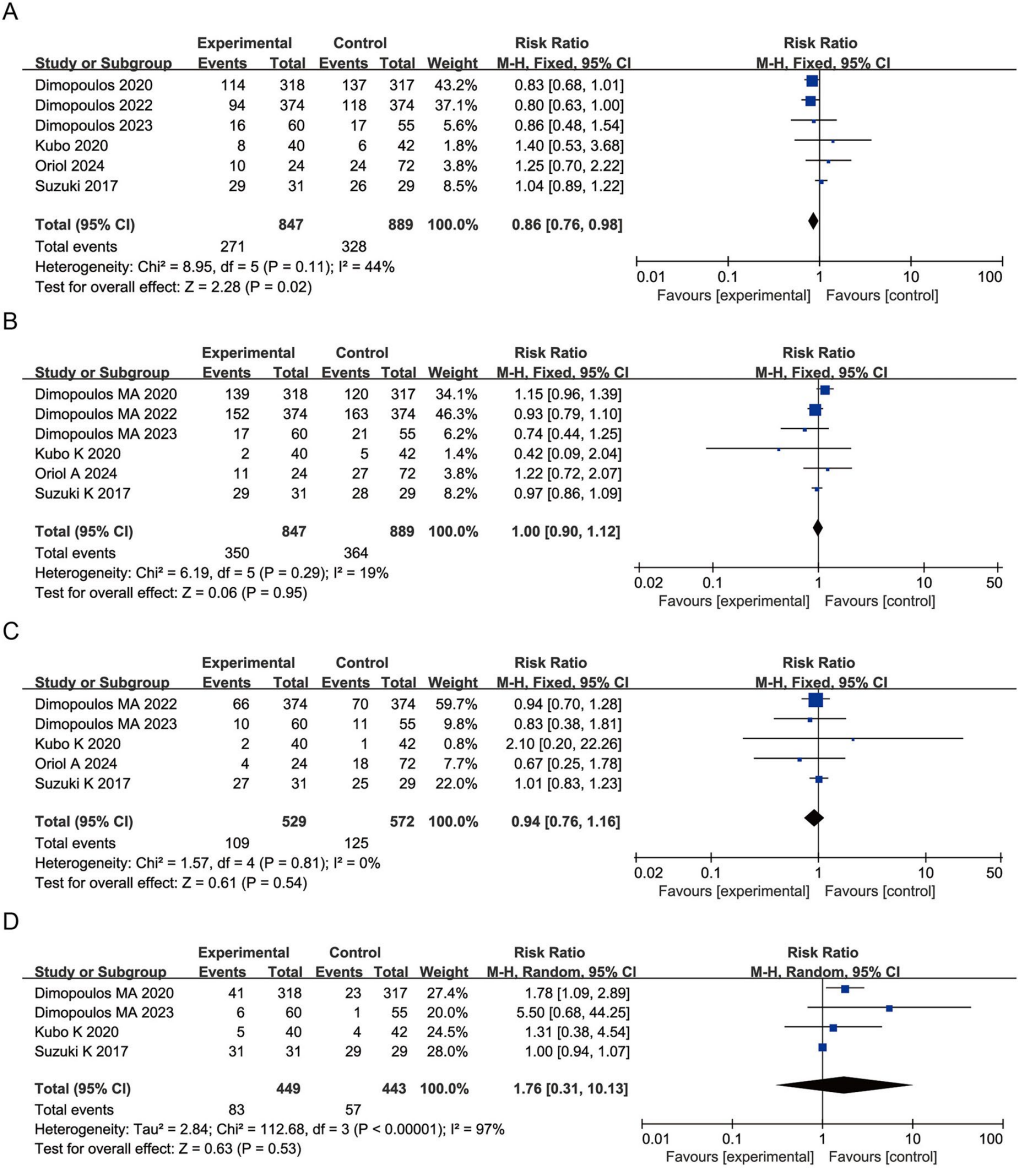
Forest plots of hematologic AEs comparing elotuzumab and control groups: (A) Neutropenia; (B) Anemia; (C) Thrombocytopenia; (D) Lymphopenia.

Elotuzumab was associated with a significantly lower risk of neutropenia compared to controls (RR = 0.86, 95% CI: 0.76–0.98; *p* = 0.02; I^2^ = 44%) (Figure 4A). No statistically significant differences were observed for anemia (RR = 1.00, 95% CI: 0.90–1.12; *p* = 0.95; I^2^ = 19%), thrombocytopenia (RR = 0.94, 95% CI: 0.76–1.16; *p* = 0.54; I^2^ = 0%), or lymphopenia (RR = 1.76, 95% CI: 0.31–10.13; *p* = 0.53; I^2^ = 97%) (Figures 4B–D). These findings indicate an association between elotuzumab-containing regimens and a lower reported incidence of neutropenia, without evidence of a significant effect on other hematologic toxicities.

### 3.6. Respiratory adverse events

**Figure 5.**
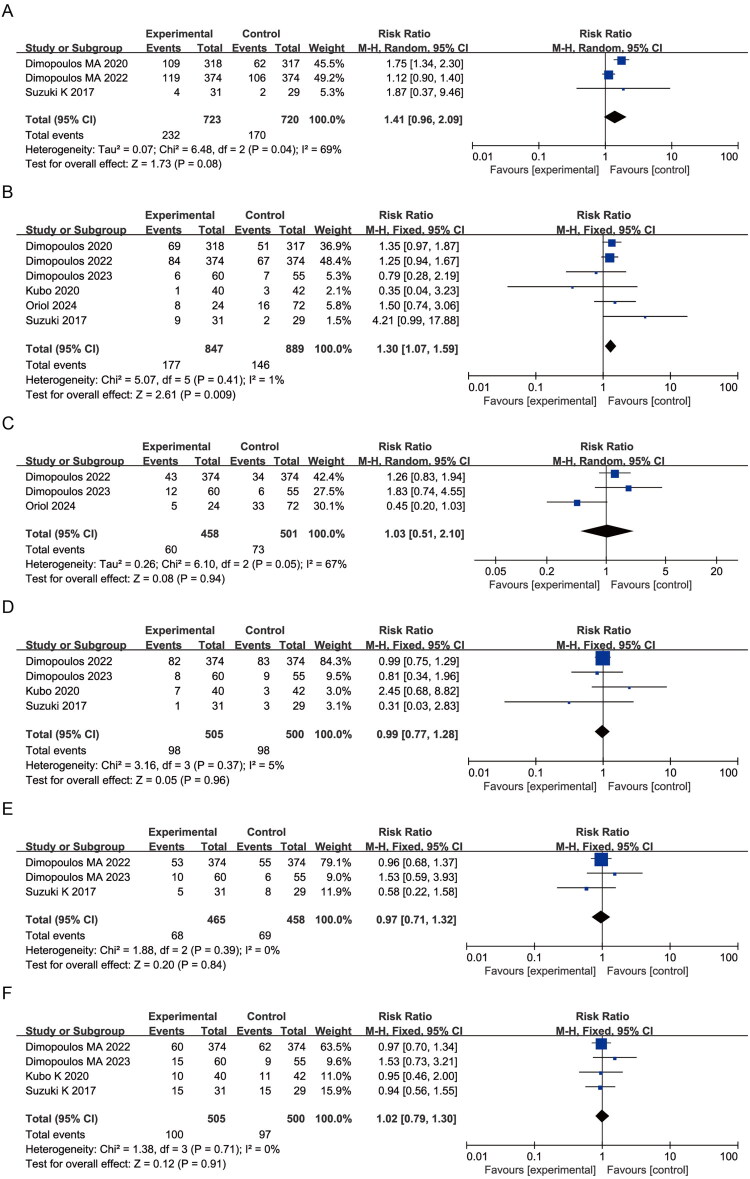
Forest plots of respiratory system AEs: (A) Cough; (B) Pneumonia; (C) Respiratory tract infection; (D) Upper respiratory tract infection; (E) Bronchitis; (F) Nasopharyngitis.

Elotuzumab-treated patients exhibited a numerically higher incidence of cough (RR = 1.41, 95% CI: 0.96–2.09; *p* = 0.08; I^2^ = 69%) and pneumonia (RR = 1.30, 95% CI: 1.07–1.59; *p* = 0.009; I^2^ = 1%) (Figure 5A,B). No significant differences were observed in respiratory tract infection (RR = 1.03, 95% CI: 0.51–2.10; *p* = 0.94; I^2^ = 67%), upper respiratory tract infection (RR = 0.99, 95% CI: 0.77–1.28; *p* = 0.96; I^2^ = 5%), bronchitis (RR = 0.97, 95% CI: 0.71–1.32; *p* = 0.84; I^2^ = 0%), or nasopharyngitis (RR = 1.02, 95% CI: 0.79–1.30; *p* = 0.91; I^2^ = 0%) (Figure 5C–F), with low or negligible heterogeneity.

### 3.7. Gastrointestinal adverse events

**Figure 6.**
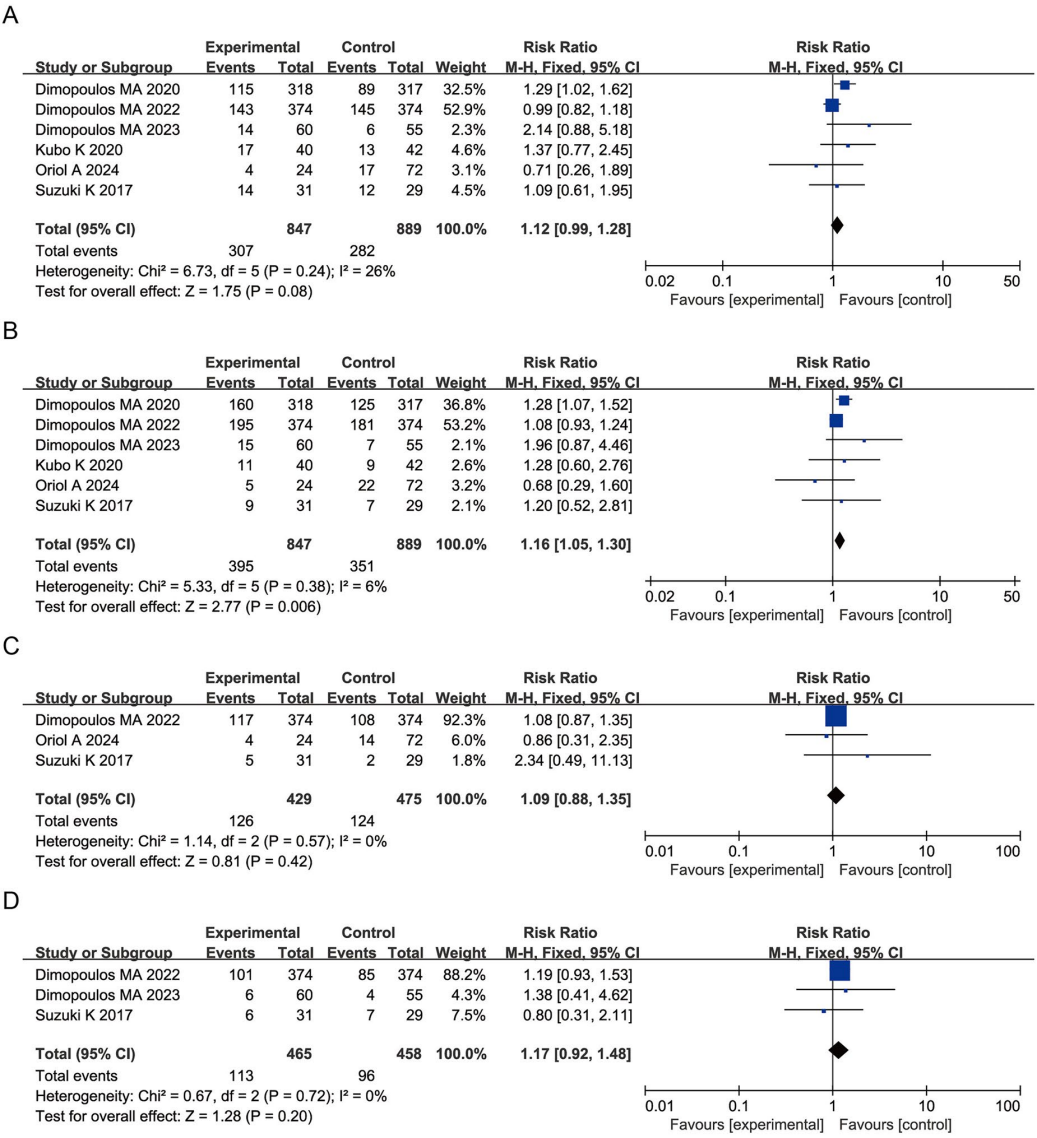
Forest plots of gastrointestinal AEs: (A) Constipation; (B) Diarrhea; (C) Nausea; (D) Decreased appetite.

A higher frequency of gastrointestinal adverse events, including constipation (RR = 1.12, 95% CI: 0.99–1.28; *p* = 0.08; I^2^ = 26%) and diarrhea (RR = 1.16, 95% CI: 1.05–1.30; *p* = 0.006; I^2^ = 6%), was observed in elotuzumab-containing regimens compared with control arms (Figure 6,B). Incidences of nausea (RR = 1.09, 95% CI: 0.88–1.35; *p* = 0.42; I^2^ = 0%) and decreased appetite (RR = 1.17, 95% CI: 0.92–1.48; *p* = 0.20; I^2^ = 0%) did not differ significantly between groups (Figure 6C,D). These findings indicate a higher reporting frequency of gastrointestinal adverse events in elotuzumab-containing regimens.

### 3.8. Musculoskeletal and connective tissue disorders

**Figure 7.**
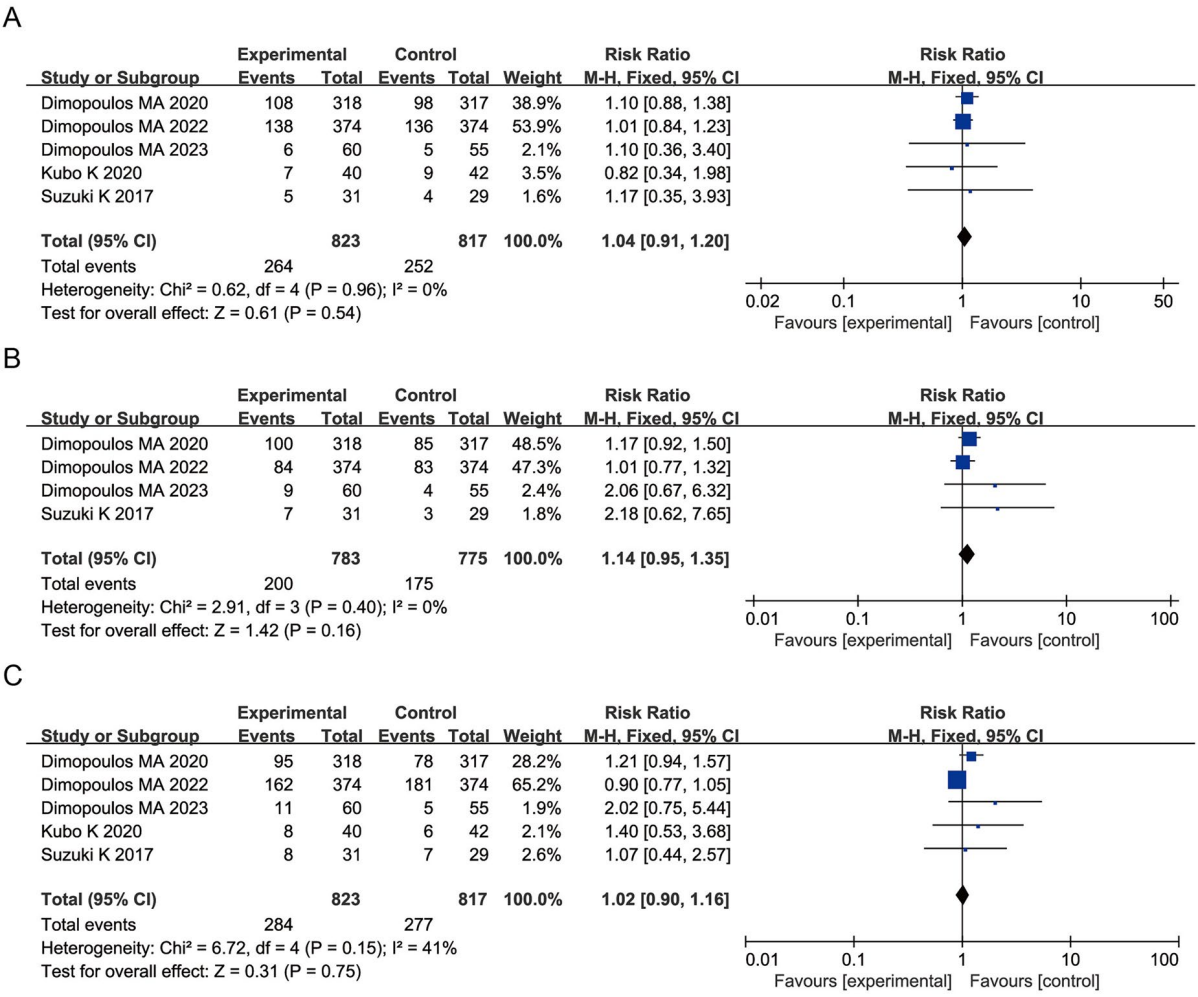
Forest plots of musculoskeletal and connective tissue AEs: (A) Back pain; (B) Muscle spasms; (C) Peripheral edema.

No statistically significant differences were observed for back pain (RR = 1.04, 95% CI: 0.91–1.20; *p* = 0.54; I^2^ = 0%), muscle spasms (RR = 1.14, 95% CI: 0.95–1.35; *p* = 0.16; I^2^ = 0%), or peripheral edema (RR = 1.02, 95% CI: 0.90–1.16; *p* = 0.75; I^2^ = 41%) (Figure 7A–C), indicating a comparable safety profile for musculoskeletal events between treatment arms.

### 3.9. Other adverse events

**Figure 8.**
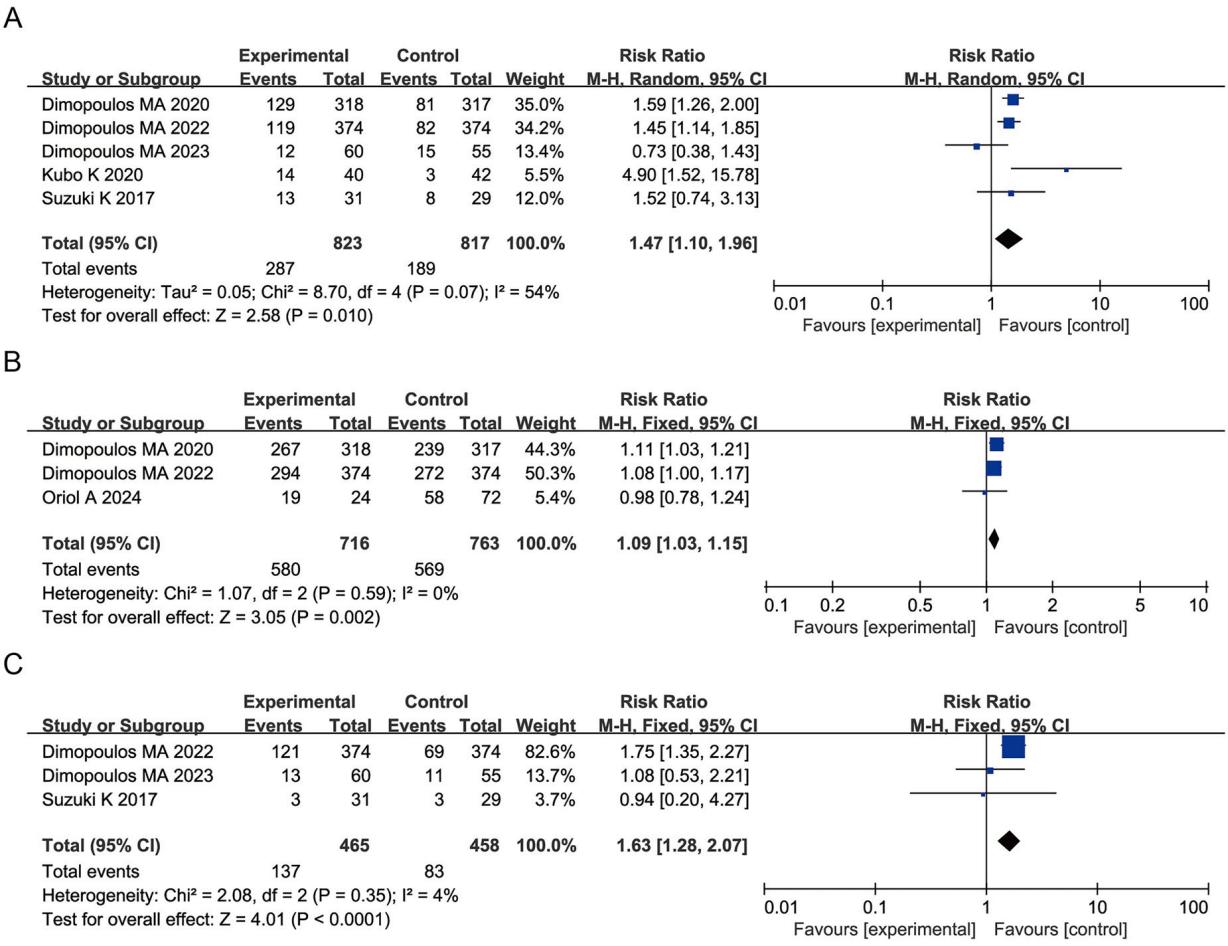
Forest plots of systemic and metabolic AEs: (A) Pyrexia; (B) Infections (any type); (C) Hyperglycemia.

**Figure 9.**
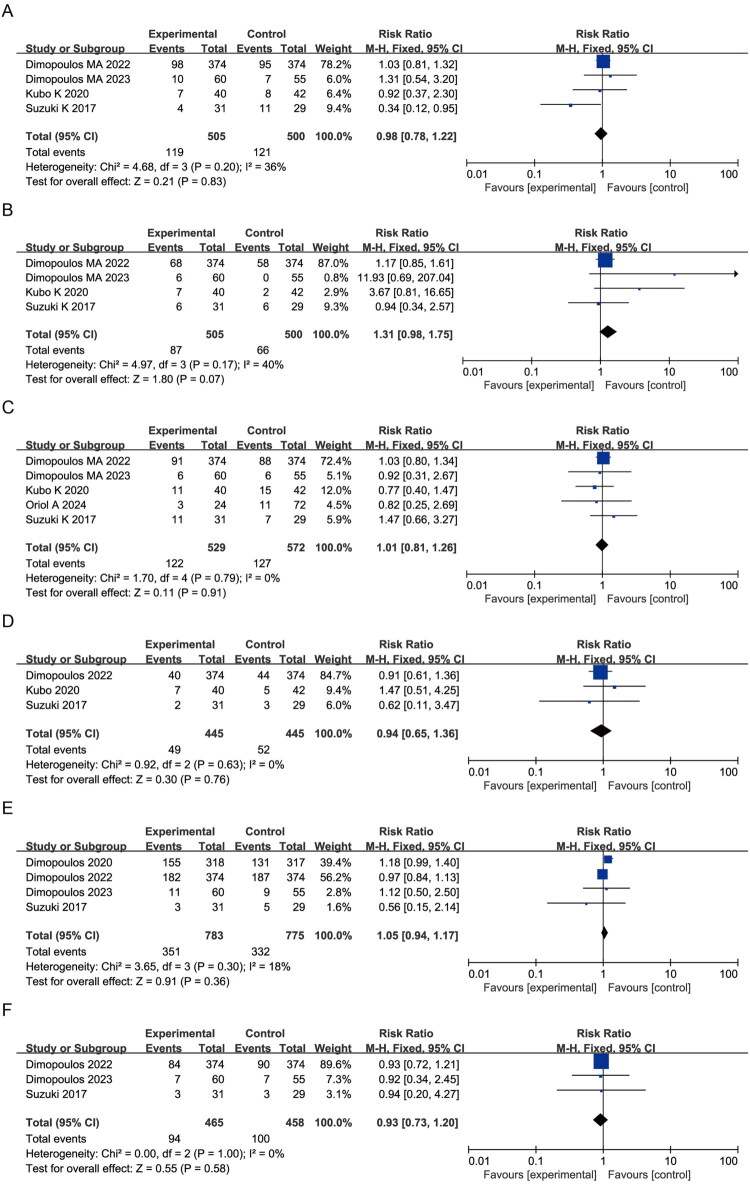
Forest plots of additional non-hematologic AEs: (A) Insomnia; (B) Cataracts; (C) Rash; (D) Pruritus; (E) Fatigue; (F) Hypokalemia.

Elotuzumab-containing regimens were associated with a higher reported frequency of pyrexia (RR = 1.47, 95% CI: 1.10–1.96; *p* = 0.010; I^2^ = 54%) and overall infections (RR = 1.09, 95% CI: 1.03–1.15; *p* = 0.002; I^2^ = 0%) (Figure 8A,B). The higher reported frequency of hyperglycemia was also significantly elevated in elotuzumab-containing regimens (RR = 1.63, 95% CI: 1.28–2.07; *p* < 0.0001; I^2^ = 4%) (Figure 8C), suggesting potential metabolic concerns associated with elotuzumab-containing regimens. No significant differences were observed for insomnia, cataract, rash, pruritus, fatigue, or hypokalemia (all *p* > 0.05) (Figure 9A–F).

### 3.10. Grade 3–4 adverse events

**Figure 10.**
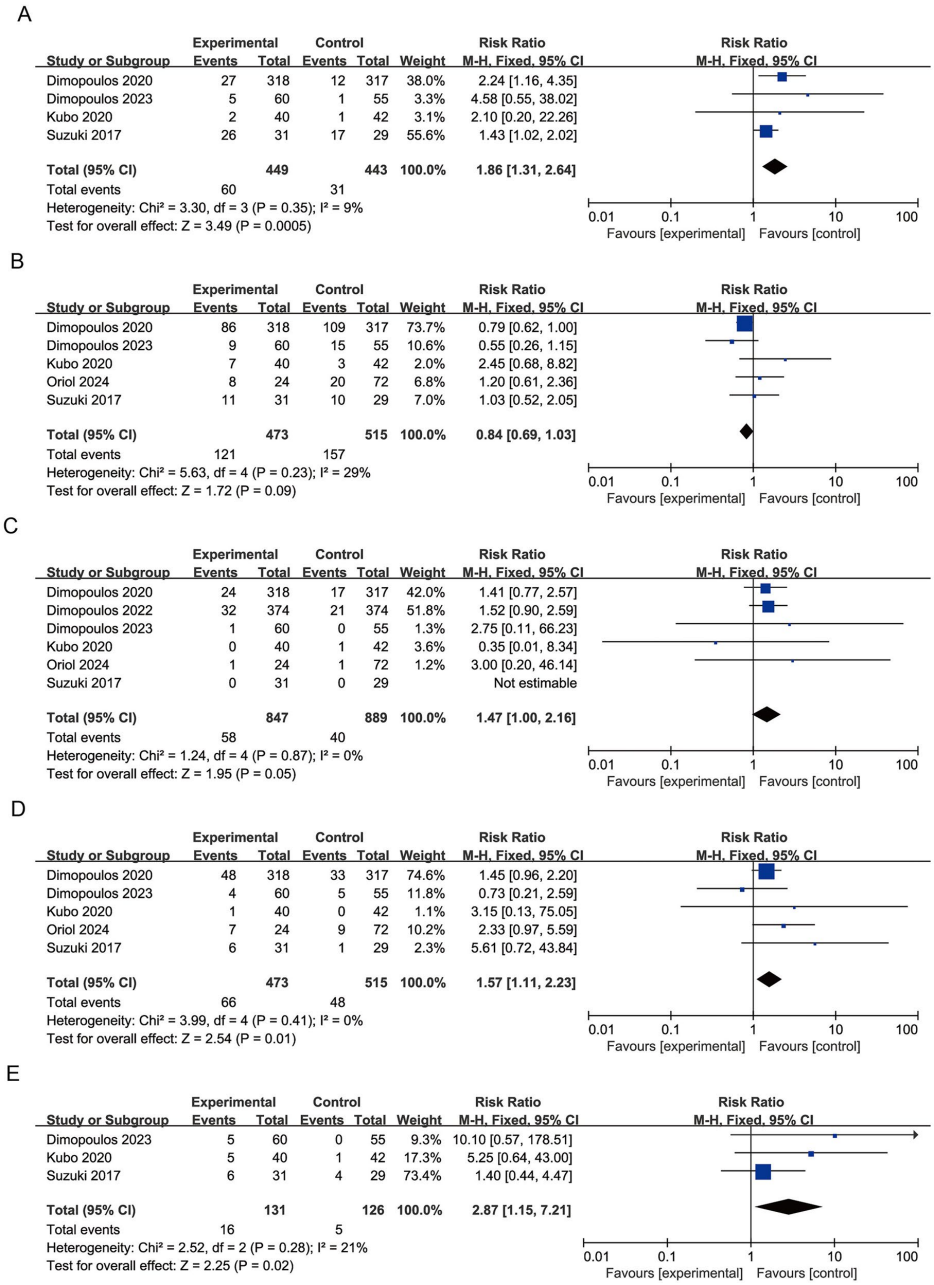
Forest plots of grade 3–4 hematologic AEs and non-hematologic AEs: (A) Lymphopenia; (B) Neutropenia; (C) Diarrhea; (D) Pneumonia; (E) Cataracts.

A meta-analysis of studies reporting grade 3–4 hematologic adverse events revealed no significant heterogeneity among the included trials. Compared with control groups, elotuzumab-containing regimens were associated with a significantly increased risk of lymphopenia (RR = 1.86, 95% CI: 1.31–2.64; *p* = 0.0005; I^2^ = 9%) (Figure 10A), highlighting the need for close monitoring. Conversely, a trend toward a reduced risk of neutropenia was observed in the elotuzumab group (RR = 0.84, 95% CI: 0.69–1.03; *p* = 0.09; I^2^ = 29%), although this did not reach statistical significance (Figure 10B).

Further analysis of grade 3–4 non-hematologic adverse events showed no heterogeneity across studies. Elotuzumab-treated patients exhibited significantly increased risks of diarrhea (RR = 1.47, 95% CI: 1.00–2.16; *p* = 0.05; I^2^ = 0%), pneumonia (RR = 1.57, 95% CI: 1.11–2.23; *p* = 0.01; I^2^ = 0%), and infections (RR = 1.32, 95% CI: 1.07–1.63; *p* = 0.009; I^2^ = 0%) compared to controls. A higher incidence of cataracts (RR = 2.87, 95% CI: 1.15–7.21; *p* = 0.02; I^2^ = 21%) was also observed. However, this finding is likely confounded by increased corticosteroid exposure in elotuzumab-containing regimens and cannot be attributed to elotuzumab itself (Figure 10C–E).

## 4. Discussion

This systematic review and meta-analysis provide the most comprehensive evaluation to date of the safety profile of elotuzumab-based regimens in patients with multiple myeloma (MM). By synthesizing data from 6 randomized controlled trials involving 1,736 participants, we found that while elotuzumab is generally well tolerated, its use is associated with a distinct pattern of adverse events (AEs), particularly affecting the respiratory, gastrointestinal, and metabolic systems.

Our analysis shows that the incidence of treatment-emergent AEs was remarkably high in both the elotuzumab and control groups, consistent with expectations in heavily pretreated MM populations receiving multi-agent therapy. Importantly, there was no statistically significant difference in the overall or grade 3–4 AE rates between groups, indicating that elotuzumab does not substantially increase the global burden of toxicity. Nevertheless, specific organ-related AEs were more frequent in patients receiving elotuzumab, highlighting the need for targeted monitoring.

One of the notable observations in this analysis was a lower reported incidence of neutropenia in patients receiving elotuzumab-containing regimens. This observation represents a consistent clinical association across IMiD-based trials. However, no mechanistic inference can be drawn from the present meta-analysis, and the finding should be interpreted as phenomenological rather than causal. Whether this observation has clinical relevance for specific patient subgroups, such as older individuals or those with limited marrow reserve, remains uncertain.

The increased risk of pneumonia in elotuzumab-treated patients should be interpreted cautiously. Differences may arise from reporting practices, patient characteristics, or treatment duration, rather than indicating a selective immunological impact of elotuzumab. Rates of upper respiratory infections and bronchitis were comparable, supporting a general rather than targeted effect.

Gastrointestinal adverse events are well-recognized background toxicities of multiple myeloma therapy, particularly in regimens containing immunomodulatory drugs and proteasome inhibitors. Therefore, the higher frequency of gastrointestinal events observed in elotuzumab-containing arms should be interpreted with caution. Importantly, patients in the elotuzumab arms generally experienced longer progression-free survival and treatment duration, which inherently increases the likelihood of cumulative adverse event reporting. While these symptoms are not uncommon in MM treatment regimens, our analysis demonstrates a modest but consistent increase in reported gastrointestinal adverse events, which may reflect cumulative toxicity associated with prolonged treatment duration rather than a specific gastrointestinal liability of elotuzumab. Accordingly, the gastrointestinal adverse events observed in this analysis should be interpreted within the context of this high background incidence.

Hyperglycemia was observed in only one included trial, and corticosteroid exposure is a likely contributor. Therefore, this signal should be interpreted cautiously as an exploratory finding, and no mechanistic inference regarding elotuzumab can be drawn. Nevertheless, clinicians may consider monitoring glucose levels, particularly in patients with preexisting metabolic conditions. While corticosteroid use likely contributes substantially to this finding, the mandatory and prolonged corticosteroid co-administration across all elotuzumab-containing regimens precludes definitive attribution of hyperglycemia to elotuzumab itself.

We also observed higher rates of pyrexia and infections in patients receiving elotuzumab. As noted above, longer treatment duration and concomitant use of immunosuppressive agents such as lenalidomide and dexamethasone may contribute to the cumulative risk of these events. These findings underscore the importance of rigorous infection monitoring, prophylaxis, and early intervention strategies when elotuzumab is incorporated into combination regimens [28,29].

Other adverse events, including musculoskeletal pain, cutaneous reactions, fatigue, and insomnia, did not differ significantly between groups [30]. Although cataracts were not significantly increased overall, a higher risk was observed in the grade 3–4 subgroup, which should be interpreted cautiously given the well-established association between long-term corticosteroid exposure and cataract formation.

Several limitations of this meta-analysis should be acknowledged. First, one trial evaluating elotuzumab with bortezomib was excluded to reduce clinical heterogeneity, as PI-based regimens differ in toxicity and are less relevant in current practice. While this limits generalizability, it improves interpretability of pooled safety estimates. Second, corticosteroids were included in all elotuzumab regimens, confounding metabolic and ocular adverse events and precluding isolation of elotuzumab-specific toxicity. Third, elotuzumab arms generally had longer treatment duration due to prolonged progression-free survival, which may contribute to cumulative reporting of infections and gastrointestinal events. Exposure-adjusted incidence rates were not uniformly reported, so analyses are based on reported TEAE frequencies. Observed differences in neutropenia should not be interpreted as evidence of a protective effect, as trial-level data do not allow mechanistic or exposure-adjusted conclusions.

## Supplementary Material

PRISMA_2020_checklist.docx

## Data Availability

The original contributions presented in the study are included in the article material. Further inquiries can be directed to the corresponding author.
